# Attenuated Psychotic Symptoms in Adolescents With Chronic Cannabis and MDMA Use

**DOI:** 10.3389/fpsyt.2021.696133

**Published:** 2022-01-21

**Authors:** Melina Wiedmann, Sören Kuitunen-Paul, Lukas A. Basedow, Veit Roessner, Yulia Golub

**Affiliations:** Department of Child and Adolescent Psychiatry, Faculty of Medicine, Technische Universität Dresden, Dresden, Germany

**Keywords:** addiction, marihuana, ecstasy, psychosis, substance abuse, THC

## Abstract

**Objectives:**

Both substance use, on the one hand, and the first signs of psychosis, on the other, commonly begin in adolescence. Adolescents with substance use disorder (SUD) frequently show recreational use of cannabis and 3,4-methylenedioxymethamphetamine (MDMA). When attenuated psychotic symptoms (APS) occur during the course of SUD, they are commonly attributed to the cannabis use, neglecting the role of other substances abused, such as MDMA in the risk of psychosis.

**Methods:**

We analyzed retrospective self-reports on APS (Prodromal Questionnaire, PQ-16) and amount of cannabis and MDMA use in *n* = 46 adolescent psychiatry outpatients with SUD. *N* = 17 (35%) individuals reported MDMA consume additional to cannabis. Furthermore, we examined the associations of APS with cannabis and MDMA use in stepwise hierarchical regressions while controlling for trauma history, birth complications and gender.

**Results:**

APS were not related to cannabis (*B* = 0.04, *p* = 0.842), but to MDMA use (*B* = 4.88, *p* = 0.001) and trauma history (*B* = 0.72, *p* = 0.001). Gender (*B* = −0.22, *p* = 0.767) and birth complications (*B* = −0.68, *p* = 0.178) were not associated with APS.

**Discussion:**

Our results indicate that MDMA use additional to cannabis use is associated with APS among adolescent SUD patients. Contrary to our expectations, we did not see an association of cannabis use and APS. We speculate that cannabis increases the risk for psychosis after a longer period of use and in combination with other risk factors, such as trauma history. Clinicians should screen for APS among SUD patients using MDMA and cannabis in order to adapt treatment plans of SUDs. Future research should validate these findings in longitudinal studies including polysubstance use and trauma history.

## Introduction

Adolescence is typically characterized by physical and psychological changes leading to impulsive and hazardous behaviors, such as increased involvement in unintentional accidents, violence, high-risk sexual behavior as well as substance use (1). Among European adolescents and young adults, aged 15–24 years, cannabis use is reported by up to 23% (Italy) and 3,4-methylenedioxymethamphetamine (MDMA) use by up to 9% (Netherlands) at least once during the past year (2).

For a part of substance using adolescents this period is also marked by the emergence of mental health problems (3), such as an onset of psychotic disorders (PDs). PDs are a group of illnesses described by the presence of unusual belief systems which do not conform to societal norms (delusions), hallucinations (particularly auditory), and disorders of thought and cognition. Both substance use, on the one hand, and the first signs of PDs, on the other, commonly begin in late adolescence and early adult life (4).

During the development of PDs non-specific and negative psychotic symptoms, which are defined as a lessening or absence of normative behaviors and functions related to motivation and interest (5), usually develop first and are followed by attenuated positive symptoms (6). 80–90% of patients suffering from schizophrenia report a period of attenuated psychotic symptoms (APS) including changes in perception, beliefs cognition, affect, mood before developing full-blown psychosis (7). In the DSM-5, APS are acknowledged as a distinct syndrome (attenuated psychotic syndrome) (8), which has been thought a risk state for later PDs and is associated with certain risk factors (9). Individuals that share a first-degree relative with a psychotic disorder (PD) are being considered at high-risk for PDs and with transition rates ranging from 18% within 6 months to 36% within 3 years (10). Additionally another even more vulnerable group for developing PDs has been defined that is considered to be at “ultra-high-risk” (7). According to (7), UHR applies young individuals (aged between 14 and 30 years old) being referred to meet one of the following criteria: (a) experiencing attenuated positive symptoms during the past year, (b) experiencing brief limited intermittent psychotic symptoms lasting no longer than 1 week and abating spontaneously or (c) being at high-risk status for PDs and showing a significant decrease in functioning during the previous year (7, 11). However, more recent definitions of UHR include substance use among other environmental factors as well since individuals with UHR for psychosis often show increased rates of substance use (6).

Also APS and PDs in adolescence are frequently associated with heavy use of cannabis and stimulants (12, 13). While up to 100% of individuals with severe and 67% for moderate amphetamine use disorder reported psychotic symptoms (OR = 35.3, 95% CI 5.1–246.5) (14), 80% of individuals with severe (OR = 26.8, 95% CI 10.3–69.6) and 79% with moderate cannabis use disorder (OR = 25.1, 95% CI 10.4–60.8) did this in the context of intoxication or withdrawal (14). Many studies indicate a dose-dependent relationship between cannabis use and risk for PDs (15, 16). A study on US- American adults showed that cannabis users (defined as at least one episode of cannabis use during a person's lifetime) were at greater risk for developing PDs (OR = 1.27, 95% CI 1.03–1.57) and schizotypal-personality disorders (OR = 2.02, 95% CI 1.69–2.42) compared to non-users (17). These results indicate that PD risk could be positively associated with higher consume rates of cannabis. However, it is difficult to draw causal conclusions from most of these studies because cannabis is often used in combination with other illicit substances, such as MDMA.

MDMA is a synthetic drug which belongs to the substance class of amphetamines and is structurally similar to the psychedelic hallucinogen mescaline (18). Dual use of MDMA and cannabis is especially prevalent in 15–16 year old adolescents, with 8–13% of whom report having used both substances within the past month (19). MDMA use has been previously shown to induce psychosis in single case studies (20–22). In rats MDMA has been shown to disrupt mechanisms of auditory sensory gating *via* its involvement in both dopaminergic and serotonergic pathways (23, 24). Sensory gating is a form of information processing where irrelevant stimuli are being ignored and is being hypothesized to be deficient in patients suffering from PDs (25). Whereas findings on the relationship between amphetamine and methamphetamine use indicate that these substances are associated with PDs (26–29), findings on MDMA are restricted to single case studies (22, 30). However, little research has been conducted on the relationship between MDMA and PDs so far and to our knowledge, there is no study that compared its relationship to simultaneous cannabis use.

In addition to substance use, other environmental factors play an important role in the development of PDs (31), such as trauma history and birth and pregnancy complications. Compared to the global population patients with PDs show higher prevalence of trauma history, such as sexual (48% females, 28% males) or physical (48% females, 50% males) child abuse (32–34). Further, birth and pregnancy complications resulting in low birth weight, gestational bleedings, emergency cesarean section and signs of asphyxia have been frequently associated with PDs (35, 36). Additionally, it is important to control for factors that have been reported to affect substance use or substance use disorders (SUDs) such as gender (37). SUDs have been shown to occur more frequently among male individuals with PDs (OR = 3.43, 95% CI 3.01–3.92) (37), indicating that it is an additional factor that influences the relationship between the risk of PD and hazardous substance use. Therefore it is important to study the relationship between these environmental risk factors and the origins of development of PDs, since transition rates from being at risk for psychosis range between 6% within two (38) and 45% within six years (39). However, it is less clear how these environmental factors, including substance use, interact with APS in adolescence. This is of great importance because PDs typically develop in adolescence and early adulthood, thus identifying risk factors could provide a base for targeted interventions.

Our study aims to investigate the relationship between self reported past year cannabis, MDMA use and APS in adolescents with SUDs while considering environmental factors and comparing the influences of cannabis and MDMA use.

We aim to examine the associations of average past year amount of cannabis, additional average past year amount of MDMA use and APS in adolescents with SUDs. We include factors such as birth complications, gender, and traumatic experience in our analyses to control for confounding influences. Furthermore, we hypothesize that 1) higher average past year amount of cannabis use is correlated with increased risk of PD in addition to other environmental factors, and that 2) higher average past year amount of additional MDMA use is correlated with higher risk of PD in addition to cannabis use and other environmental factors.

## Methods

### Participants

Between November 2017 and January 2020, *n* = 198 treatment-seeking adolescents (aged 13–18) with SUDs and their legal guardians gave written informed consent to participate in the study. We excluded *n* = 113 (57%) adolescents who did not return all required questionnaires, *n* = 17 with consumption of substances other than nicotine, alcohol, cannabis and MDMA during the last month and year, i.e., amphetamine, methamphetamines, cocaine, hallucinogens or solvent sniffing and *n* = 7 who left too many single items unanswered (>20% of items). In order to focus solely on APS, participants with psychotic disorders (*n* = 7 with diagnoses F1X.5, F1X.7, F30.2 and F2X) were excluded from the sample. Participants with diagnoses of other psychiatric disorders were not excluded. Further, participants who reported 0 cannabis exposure during previous 12 months (*n* = 8) were excluded as well. In the final sample of *n* = 46, the mean age was 16.19 (*SD* = 1.23) and 46% were female, see Table 1. The final sample consisted of SUD patients all with past year cannabis use, and partly of additional MDMA users (*n* = 17). No individual reported MDMA use only.

**Table 1:** Sample characteristics of cannabis and additional MDMA users.

**Characteristics**	**Total (*n* = 46)**	**Cannabis users without**	**Cannabis users with additional**	**Group comparison**		
		**MDMA use (*n* = 29)**	**MDMA use (*n* = 17)**	**Test statistic**	***p-value***	**Effect size**
				**(*df*)**		
**Socioeconomic data**						
Female gender (%)^a^	21 (46)	9 (31)	12 (71)	χ^2^(1,46) = 6.76	0.009	ϕ = 0.38
Age (SD)^b^	16.19 (1.23)	16.39 (1.16)	15.84 (1.29)	*F*_(1,46)_ = 2.17	0.148	$\eta_{\text{part}}^{2}$ = 0.05
Drop-out from school^a^ (%)	4 (12%)	1 (4%)	3 (28%)	χ^2^(1,34) = 3.77	0.052	ϕ = 0.33
Living with both parents^a^ (%)	6 (19%)	5 (23%)	1 (10%)	χ^2^(1,32) = 0.73	0.393	ϕ = −0.15
Medical comorbidity^a^ (%)	12 (35%)	7 (30%)	5 (55%)	χ^2^(1,34) = 0.74	0.391	ϕ = −0.15
APS (SD)^b^	4.08 (3.55)	2.99 (2.56)	5.93 (4.26)	*F*_(1,46)_ = 8.60	0.005	$\eta_{\text{part}}^{2}$ = 0.16
**Average daily amount of past year substance use (SD)**						
Cannabis (in grams)^b^	1.48 (1.86)	1.17 (1.48)	2.00 (2.33)	*F*_(1,46)_ = 2.19	0.146	$\eta_{\text{part}}^{2}$ = 0.05
MDMA additional to cannabis (in pills)^b^	0.23 (0.41)	-	0.62 (0.46)			–
Trauma history^b^	1.96 (2.00)	1.75 (2.05)	2.33 (1.91)	*F*_(1,46)_ = 0.89	0.350	$\eta_{\text{part}}^{2}$ = 0.02
Birth complications^c^	0.65 (0.98)	0.62 (0.93)	0.71 (1.07)	*F*_(1,46)_ > 0.09	0.762	$\eta_{\text{part}}^{2}$ = 0.02
**Co-occurring psychiatric disorders (DSM-V, %)**						
Alcohol use disorder^b^	12 (39)^d^	8 (44)^e^	4 (31)^f^	χ^2^(1,46) = 0.60	0.440	ϕ = −0.14
Affective disorder^b^	13 (42)^d^	6 (33)^e^	7 (54)^f^	χ^2^(1,46) = 1.30	0.253	ϕ = 0.21
Anxiety disorders^b^	11 (36)^d^	5 (28)^e^	6 (46)^f^	χ^2^(1,46) = 1.11	0.291	ϕ = 0.19
Post traumatic stress disorder^b^	5 (16)^d^	2 (11)^e^	3 (23)^f^	χ^2^(1,46) = 0.80	0.371	ϕ = 0.16
Conduct disorder^b^	9 (29)^d^	4 (22)^e^	5 (39)^f^	χ^2^(1,46) = 0.97	0.326	ϕ = 0.18

*MDMA, 3,4-Methylenedioxymethamphetamine*.

a *Categorical variable, group differences were tested with Chi-Squared Tests, effect sizes are expressed as Phi (ϕ)*.

b *Continuous variable, group differences are tested with MANOVA, effect sizes are expressed as partial eta squared (η^2^_part_)*.

c *Ordinal variable (0 = not present, 1 = present either before/during/after birth, 2/3 = present at two/three of these time points), group differences are tested with ANOVA, effect sizes are expressed as partial eta squared (η^2^_part_)*.

d *Not available for n = 15 (33%)*.

e *Not available for n = 11 (38%)*.

f *Not available n = 4 (24%). Substance use variables display the amount of self-rated cannabis or MDMA per calendar day during the last 12 months*.

### Procedure

Adolescents and their legal guardians who applied to the outpatient clinic for adolescent substance abuse, University Hospital C. G. Carus Dresden, Germany, were informed about the study in a first consultation appointment at the clinic. We embedded all data collection into the standard diagnostic procedures. All questionnaires were handed out at the first consultation before any intervention started. Substance use questionnaires and diagnostic interviews were performed during the same appointment. All interviews were conducted by clinically trained and supervised staff members (psychologists, and child and adolescent psychiatrists). All procedures were conducted in accordance with the Declaration of Helsinki and, were approved by the Institutional Review Board of the University Hospital C. G. Carus Dresden (EK 66022018) and registered at clinicaltrials.gov (NCT03444974). Both patients and their legal guardians agreed to participate by written consent after a comprehensive verbal and written information.

### Measures

#### Attenuated Psychotic Symptoms

The Prodromal Questionnaire (PQ) (40) is a self-report screening questionnaire assessing the presence of APS on a two-point scale (true/false) and subjective load on a four-point Likert scale ranging from 0 (“not at all”) to 3 (“very much”). We used the German 16-Item short version (PQ-16) (41) in order to make the process of data collection less time consuming for patients. The total score was calculated by the sum of positively answered symptoms (“true”) ranging from 0 to 16. We did not use values of subjective load for the current analysis. The PQ-16 shows acceptable internal consistency with a Cronbach's alpha for its total score of 0.77 (41). Its total score is also correlated (*r* = 0.57, *p* < 0.001) with the Comprehensive Assessment of At-Risk Mental States (CAARMS) score, which identifies the symptoms of a first psychotic episode with good to excellent reliability (*r* = 0.85) (11). The PQ-16 questionnaire contains two major subscales: positive (14 items) and negative symptoms (2 items). Positive items contain two subscales: Unusual thought content/delusional ideas (5 items) and perceptual abnormalities/hallucinations (9 items). Negative symptoms assess excessive social anxiety and avolition with one item each. Total scores of six or more positively answered items fulfill criteria for clinical high-risk status (CHR).

#### Past Year Substance Use

Participants gave information about their use of nicotine, alcohol, cannabis, MDMA, amphetamine, methamphetamine, cocaine, hallucinogens, ketamine, opiates, other substances and internet use in a personal interview. We assessed: age of first use (“*How old were you when you first used Cannabis?”*), experimental drug use (“*After this time when you first used Cannabis, did you continue to use Cannabis?”*), days and dose for previous month and year (e.g., “*On how many days during the previous month did you use Cannabis and how much Cannabis did you use on an average day?”* or “*During the last year, on how many days per month did you averagely use Cannabis and how much Cannabis did you use on an average day?”*). The variable “average amount of past year cannabis use” and “average amount of past year MDMA use additional to cannabis” denotes the average daily amount of used cannabis (in gram) or MDMA (in pills) per calendar day during the last year previous to the date that individuals presented themselves at the clinic and is scaled in an interval level.

#### Trauma History

We used the University of California at Los Angeles Post Traumatic Stress Disorder Reaction Index for DSM-IV (UCLA PTSD) (42), German version (43) instrument to assess trauma history. The UCLA PTSD is a self-report questionnaire that assesses exposure to traumatic events and Post-Traumatic-Stress-Disorder (PTSD) symptoms in young children and adolescents. With reported values of 0.88–0.91 for Cronbach's alpha (44), the UCLA PTSD shows good to excellent internal consistency.

#### Pregnancy and Birth Complications

We asked the subjects' parents or caregivers *via* questionnaire whether there were any kinds of pregnancy and/or birth complications (rating with yes/no, including irregularities during pregnancy, birth itself and short time after birth).

#### ICD-10 Diagnoses

We used the Mini-International Neuropsychiatric Interview for Children and Adolescents (MINI-KID) (45) to assess co-occurring psychiatric disorders. The MINI-KID is a structured interview for DSM-V and ICD-10 psychiatric disorder in children and adolescents with substantial to almost perfect interrater and test-retest reliability (*k* = 0.64–1.00) (46).

#### Socioeconomic Data

We collected socioeconomic data from parents or caregivers at the first consultation appointment. Data concerning drop up from school *(“Did the child drop out from school?”*), living with both parents in same household *(“Does the child live with both parents in the same household?”*) and presence of medical comorbidity *(“Have any former medical or neurological ever diseased been diagnosed?”*) were used for the current analysis.

#### Data Analysis

We replaced missing values within each regression model using single imputation expectation-maximization for the following variables: trauma history (4% values were missing), birth complications (13%), cannabis use (36%), and MDMA use (36%). If participants did not fill in four or more items (>20%), they were excluded from the analyses (*n* = 7). For those with less missing values than four, the mean value of total score was used to replace missing values for single items. All analyses were performed with IBM SPSS Statistics 25.0 (IBM SPSS Statistics for Windows, Version 25.0). We calculated Pearson's correlation coefficients *r* between average amount of past year cannabis and MDMA use per day, trauma history, gender and presence of birth complications and APS. The level of significance was defined as *p* < 0.05 (two-tailed). We performed a bias-corrected and accelerated bootstrapped (BCa-method, *N* = 1,000 repetitions) regression analysis *via* enter method because data were not normally distributed. Model 1 predicted the number of APS (PQ-16 sum score) by control variables (trauma history, gender and birth complications). Model 2 added average amount of past year cannabis use. Further, model 3 included average amount of past year MDMA use additional to cannabis. Effect sizes were classified into small effects (|*r* | ≥ 0.10, | η^2^_part_ | ≥ 0.01, |φ | ≥ 0.10), medium effects (|*r* | ≥ 0.30, | η^2^_part_ | ≥ 0.06, |φ | ≥ 0.30), and large effects (|*r* | ≥ 0.50, | η^2^_part_ | ≥ 0.14, |φ | ≥ 0.50) (47). Additional test of multicollinearity were performed. The cut off values for tolerance of <0.10 and > 5 for VIF would hereby indicate high intercorrelations among two or more independent variables which would need to be avoided since this can cause misleading results (48). We further performed sensitivity analyses with variables of co-occurring psychiatric disorders as additional predictors in model 4 (Supplementary Table S1).

## Results

### Sample Characteristics

The sample consisted of *n* = 46 participants who reported regular use of MDMA and cannabis use during the previous year. Descriptive statistics in Table 1 show that the proportion of females was higher in the group of subjects who used cannabis and MDMA compared to those who used only cannabis [χ^2^_(1,46)_ = 6.76, *p* = 0.009]. Individuals with additional MDMA use reported more APS [*F*_(1,46)_ = 8.60, *p* = 0.005, η^2^_part_ = 0.16]. Twelve Percentage of the sample was reported to have dropped out from school, with dual users showing a tendency to higher drop-out rates [χ^2^_(1,34)_ = 3.77, *p* = 0.052, ϕ = 0.33]. 19% lived with both parents in the same household, 23% of the cannabis only group and 10% of the dual user group, which was a non-significant large difference [χ^2^_(1,32)_ = 0.73, *p* = 0.393, ϕ = −0.15]. Duals users also showed a tendency toward higher rates of medical comorbidity [55%, χ^2^_(1,34)_ = 0.74, *p* = 0.391, ϕ = −0.15].

### Associations Between APS and Model Predictors

**Figure 1:**
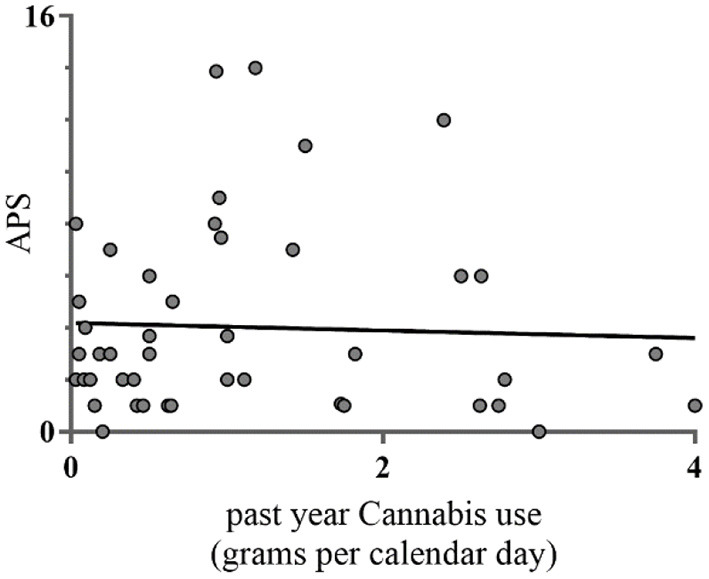
Pearson correlations between past year cannabis use and APS (*n* = 46). This figure denotes the relationship between average amount of past year cannabis use and number of APS measured with the Prodromal Questionnaire (PQ16). Cannabis use on the x-axis denotes the average amount of cannabis in grams per calendar day during past year.

**Figure 2:**
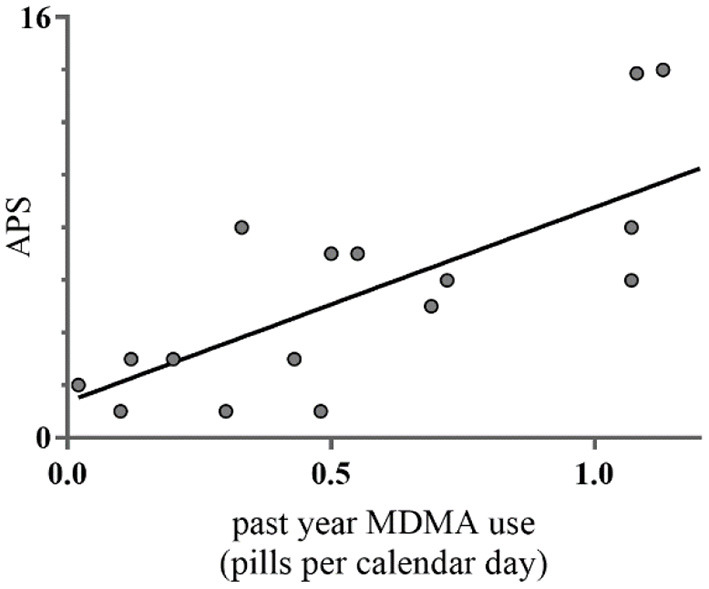
Pearson correlations between past year MDMA use additional to cannabis and APS (*n* = 17). This figure denotes the relationship between average amount of past year MDMA use additional to cannabis and number of APS measured with the Prodromal Questionnaire (PQ16). MDMA use on the x-axis denotes the average amount of MDMA in pills per calendar day during past year.

Average amount of past year MDMA use additional to cannabis use showed the strongest relationship with APS (*r* = 0.68, *p* < 0.001; see Table 2). Females showed a tendency to have higher amount of additional MDMA use (*r* = 0.40, *p* = 0.006). The average amount of past year cannabis use (*r* = −0.09, *p* = 0.532) was not associated with APS. Cannabis and additional MDMA use were not associated with each other (*r* = 11, *p* = 0.477). Trauma history (*r* = 0.63, *p* < 0.001) showed the strongest relationship with APS among control variables. Cannabis use and birth complications showed a strong correlation with each other (*r* = 0.71, *p* < 0.001). Distributions of APS for cannabis and additional MDMA use are displayed in Figures 1, 2.

**Table 2:** Summary of Pearson correlations *r* (*p*-value) of self-rated APS, control variables, cannabis and MDMA use and co-occurring psychiatric disorders (*n* = 46).

		**1**	**2**	**3**	**4**	**5**	**6**	**7**	**8**	**9**	**10**	**11**
1	APS^a^											
2	Birth complications^c^	−0.27 (0.065)										
3	Female gender^b^	0.25 (0.098)	0.08 (0.613)									
4	Trauma history^a^	0.63 (<0.001)*	−0.20 (0.183)	0.16 (0.282)								
5	Average amount of past year cannabis use (in grams)^a^	−0.09 (0.532)	0.71 (<0.001)*	<0.01 (0.985)	−0.09 (0.536)							
6	Average amount of past year additional MDMA use (in pills)^a^	0.68 (<0.001)*	−0.02 (0.918)	0.40 (0.006)	0.31 (0.035)	0.11 (0.477)						
7	Alcohol Use Disorder^b^	−0.09 (0.643)	0.06 (0.765)	0.16 (0.395)	0.15 (0.426)	0.04 (0.85)	−0.19 (0.317)					
8	Affective disorder^b^	0.52 (0.003)	−0.02 (0.918)	0.75 (<0.001)*	0.53 (0.002)	−0.02 (0.898)	0.35 (0.057)	0.13 (0.486)				
9	Anxiety disorders^b^	0.40 (0.026)	−0.21 (0.249)	0.50 (0.005)	0.55 (0.001)	−0.20 (0.269)	0.33 (0.071)	0.10 (0.582)	0.60 (<0.001)*			
10	Post Traumatic Stress Disorder^b^	0.37 (0.039)	−0.19 (0.294)	0.45 (0.011)	0.57 (0.001)	−0.11 (0.549)	0.10 (0.602)	0.19 (0.302)	0.52 (0.003)	0.59 (<0.001)*		
11	Conduct Disorder^b^	0.12 (0.506)	0.13 (0.473)	0.23 (0.205)	0.23 (0.206)	0.20 (0.27)	0.06 (0.745)	0.22 (0.232)	0.03 (0.862)	0.12 (0.521)	0.30 (0.102)	

*APS, attenuated psychotic symptoms measured by the total score of the German 16 Item short version of the Prodromal Questionnaire. MDMA, 3,4-methylenedioxymethamphetamine*.

a *Continuous variable*.

b *Categorical variable*.

c Ordinal variable (0 = not present, 1 = present either before/during/after birth, 2/3 = present at two/three of these time points). The p-value was adjusted according to the Bonferroni-procedure to p < 9.1^*^e−4 (*). Substance use variables display the amount of self-rated cannabis or MDMA per calendar day during the last 12 months.

### Hierarchical Multiple Regression Analysis

Model 2 included average amount of past year cannabis use to control variables (Model 1) and was not associated with APS (*B* = 0.31, *p* = 0.155; see Table 3). Total variance explained in APS remained at 39% with adding cannabis use to control variables in this step (*F*_3,42_ = 8.18, *p* < 0.001). Furthermore, including average amount of past year additional MDMA use in Model 3 (*B* = 4.88, *p* = 0.001) increased the explained variance in APS to 65% (*F*_5,40_ = 17.38, *p* < 0.001). The association between trauma history and APS remained significant after including cannabis and MDMA use additional to cannabis (*B* = 0.72, *p* = 0.001). The negative correlation between birth complications and APS in Model 1 and 2 was reduced to non-significance (*B* = −0.68, *p* = 0.1780) after controlling for past year additional MDMA use in Model 3.

**Table 3:** Summary of hierarchical multiple regression analysis for variables of trauma history, birth complications, gender, cannabis and additional MDMA use measures predicting APS (*n* = 46).

**Variable**	**Model 1**			**Model 2**			**Model 3**		
	**Standardized**	***p*-value^**a**^**	**95% CI^**a**^**	**Standardized**	***p*-value^**a**^**	**95% CI^**a**^**	**Standardized**	***p*-value^**a**^**	**95% CI^**a**^**
	**Regression**			**Regression**			**Regression**		
	**Coefficient *B*^a^**			**Coefficient *B*^a^**			**Coefficient *B*^a^**		
**Control variables**									
Birth complications	−0.60	0.045	(−1.26, −0.11)	−1.04	0.040	(−2.40, −0.31)	−0.68	0.178	(−1.91, 0.34)
Gender	1.18	0.182	(−0.81, 2.70)	1.25	0.163	(−0.68, 2.86)	−0.22	0.767	(−1.60, 1.17)
Trauma history	0.97	0.001	(0.58, 1.50)	0.96	0.001	(0.56, 1.49)	0.72	0.001	(0.42, 1.09)
**Average amount of past year substance use**									
Cannabis use (in grams) cannabis time since fist use cannabis time since fist use				0.31	0.155	(−0.13, 1.20)	0.04	0.842	(−0.75, 0.64)
MDMA use additional to cannabis (in pills)							4.88	0.001	(2.50, 6.76)
**Test statistics**									
Corrected *R^2^*	0.39			0.39			0.65		
*F* (*p*-value)	10.57 (<0.001)			8.18 (<0.001)			17.38 (<0.001)		
Δ*R^2^*				0.01			0.24		
Δ*F*				1.00 (0.323)			30.56 (<0.001)		

*MDMA, 3,4-Methylenedioxymethamphetamine. CI, confidence interval*.

a *Boostrapped values. Substance use variables display the amount of self-rated cannabis or MDMA per calendar day during the last 12 months*.

Tests of multicollinearity indicated that multicollinearity was not of concern (trauma history, tolerance = 0.86, VIF = 1.16; birth complications, tolerance = 0.46, VIF = 2.19; gender, tolerance = 0.81, VIF = 1.23; cannabis use, tolerance = 0.47, VIF = 2.14; MDMA use, tolerance = 0.75, VIF = 1.34).

Sensitivity analyses, which were added co-occurring psychiatric disorders to the regression model 3, revealed that the association between MDMA use and increased APS was not affected by co-occurring psychiatric disorders, see Supplementary Table S1.

## Discussion

We aimed to examine the association between self-reported average amount of past year cannabis use, MDMA use and self-rated APS among adolescents with SUDs. We controlled for environmental factors, such as trauma history, birth or pregnancy complications and gender. Contrary to our predictions, the average amount of past year cannabis use was not associated with APS. At the same time, past year MDMA use was positively associated with APS.

Our findings indicate a dose-response relationship between APS and MDMA use additional to cannabis use. Similar results were obtained by (49) who examined the additive effects of MDMA to cannabis use on APS. In their sample of young adult military conscripts in Turkey (age of individuals with cannabis and MDMA use *M* = 20.47, *SD* = 0.79 years) MDMA use showed weak to moderate positive correlations with APS. MDMA free periods were negatively correlated with APS, seemingly confirming an assumption from single-case studies that MDMA is able to induce acute APS (20, 22, 30). The associations between MDMA use and increased risk for APS were not affected by presence of co-occurring psychiatric disorders. Our results revealed a tendency toward higher APS and in affective and anxiety disorders and PTSD. This is in line with previous studies indicating that adolescent with clinical high risk for psychosis show increased psychiatric comorbidity (50, 51).

Contrary to previous studies and meta-analyses showing a strong association between cannabis use and increased risk of PDs (15, 52), we failed to find an association between cannabis use and APS. However, most of these studies examined the relationship between cannabis use and APS or psychosis in adult samples, whereas our sample consisted of adolescents. A study in US-American non-help seeking adolescents [*M* = 16.90, *SD* = 1.85; (53)] found no association between cannabis use itself (after controlling for use of other substances) and APS or psychosis. In the follow up analysis of this study (53), cannabis among other substances was associated with APS, but this was reduced to a trend after correction for multiple testing. This indicates that cannabis may only contribute to developing APS among other risk factors and therefore might not or only weakly present as a risk factor for APS in epidemiological studies. Further, another possible explanation could be that the effects of cannabis use on APS cumulate during persistent use in adolescents and may emerge later in development which is supported by another study (54).

Additionally, there are studies that reveal mixed findings on the relationship between cannabis use and APS when considering polysubstance use. In another study (55) poly-drug use (psychomotor stimulants, MDMA included) accounted for the link between cannabis use and APS in an adult sample. Unfortunately, the associations between additional MDMA use without use of other stimulants and APS were not analyzed, probably due to small sample size of individuals only using MDMA. However, these findings further strengthen the hypothesis that stimulant use (including MDMA) plays an important role in the development of APS and at least partly accounts for the relationship between cannabis use and psychosis in poly-drug users.

Another factor that might affect the relationship between cannabis use and APS is the experience of traumatic events, especially in childhood. The association between cannabis use and APS has been shown to interact with history of childhood trauma and abuse (56). In this study among young adults, cannabis use in individuals with genetic predisposition for psychosis was only associated with APS when having a history of childhood abuse (56). These results indicate that trauma history could be a crucial factor in the interaction with genetic predisposition for a development of psychosis among cannabis using adolescents or young adults.

Additionally, there is evidence that trauma history and cannabis use are risk factors for PDs, not only in genetically predisposed individuals (31). Cumulative effects of trauma history and cannabis use were shown to heighten persistence rates of APS (57, 58) and therefore interfere with the development of psychosis. Collecting data from three large longitudinal studies (57), found that persistence rates of baseline APS were higher with greater exposure to cannabis use or childhood trauma. Together, the effects of trauma history and its neurophysiological changes (59), in combination with adolescent substance use, could contribute to a higher risk of developing psychosis (60).

However, cannabis use and trauma history do not correlate with each other in our sample, whereas trauma history and MDMA use additional to cannabis were correlated. This association between trauma history and MDMA use is worth noting because MDMA has its origins as a therapeutic agent trauma focused psychotherapy. However, trauma history and additional MDMA use each were independently associated with APS. Therefore, trauma history might account for the relationship between cannabis use and APS but does not account for the relationship between additional MDMA use and APS.

MDMA and cannabis use have been associated with increased serotonin receptor sensitivity and this could increase the risk of psychosis. MDMA increases dopamine and serotonin activity in the striatum, hippocampus, prefrontal cortex and midbrain (61, 62). Further, single doses of MDMA injection lead to altered serotonin receptor response up to 6 months in rats (63) and cause serotonin receptor hypersensitivity (64). Additionally, serotonin receptor sensitization has been observed after chronic Δ9 –tetrahydrocannabinol (THC, the main psychoactive compound of cannabis) exposure in rats (65). An upregulated serotonin system is hypothesized to lead to a chain reaction involving the glutamate and dopamine system in mesolimbic areas and finally result in APS *via* an altered salience system (66). Experiencing symptoms, such as delusions or hallucinations, might represent the individuals effort to make sense of it as aberrantly salient experiences or internal representations (67).

Although we cannot draw any causal inferences from our results, we speculate that cannabis use and additional MDMA use affect the development of APS differently. The effects of heavy cannabis use during adolescence on APS might only emerge later in adulthood and might only do so in combination with other risk factors, e.g., trauma history or genetic predisposition. MDMA use on the other hand, might be a risk factor for APS independent of cannabis use and trauma history. Our results indicate that its effects already seem to be present in adolescence. Therefore, adolescent MDMA use could be a risk factor for psychosis later in life, after APS manifest. However, future research should apply longitudinal designs including these risk factors to examine the long-term effects of recreational cannabis and MDMA use and trauma history.

According to our results, the association between MDMA use additional to cannabis and APS, leads us to conclude that adolescents with such a pattern of substance use might be especially at risk for PDs. Screening and regular checks for APS among MDMA using adolescents could enable medical practitioners to identify the need for and provide medical support at an early stage. Medical treatment of symptoms of psychosis needs to be implemented in treatment plans of SUDs and could be crucial for its success because of their severe impairment of functioning associated with psychosis and risk for injuries (68).

## Limitations

As there are several limitations, our results must be interpreted with caution. First, the sample size for computing hierarchical regression with five factors would need a greater sample size to detect small effects. However, we focused on choosing a sample that is as homogenous as possible concerning types of used substances resulting in exclusion of many subjects that were using stimulants other than MDMA on a regular basis. In consideration of the fact that research on the effects of MDMA use in addition to cannabis is sparse and studies on SUDs in adolescents often struggle with small sample sizes due to weak compliance, we believe that our results can still give a valuable contribution to research on substance use and PDs. However, replication in larger samples with higher statistical power is needed to confirm these findings.

Second, our analyses are cross-sectional. Therefore, we are not able to conclude whether or not cannabis or additional MDMA use is a causal factor for APS, since we have no information about preexisting APS. Furthermore, we cannot say whether APS in our study are permanent and actually lead to psychosis, due to the cross sectional design of our study. It is possible that in some individuals of our sample PS are due to acute (withdrawal) effects of cannabis/MDMA and might remit after abstinence. Therefore, longitudinal studies are needed to draw causal conclusions between cannabis/MDMA use and APS and further examine the duration of APS associated with MDMA use.

Third, we were not able to include participants that used MDMA exclusively. Therefore, we could not investigate the effect of MDMA use on APS independently from additional cannabis consume. However, a previous study found that most MDMA users also use cannabis on a regular basis (69), which indicates that examining the associations between MDMA only and APS might refer to a small population of users. Therefore, we think that our study design is in line with clinical observations indicating seldom MDMA abuse as an only used substance. Future studies should focus on long-term effects of cannabis and additional MDMA use on psychosis and differentiate between cannabis use without additional substance use, MDMA use without additional substance use and dual use of both substances. Further, it remains unclear if the association between additional MDMA use and APS in our study can be traced back to polysubstance use in general, which is up to future studies to examine.

Fourth, the instrument for assessing APS did not specify the period in which APS occurred. Therefore, we cannot conclude that various APS occurred in a short period, which would implicate a more severe form of APS and increased risk for psychosis, e.g., when symptoms occur during a period of 2 week (see acute transient psychotic disorder; WHO, International Statistical Classification of Diseases and Related Health Problems, ICD-10).

Finally, we were not able to record objective measures of substance use across our population. Rating of substance use might be underreported by individuals due to recall biases and social stigma. However, these are influences that should affect all individuals equally which keeps the effect constant over both groups and cannot lead to the observed differences across the groups. This is an important topic which is why we currently work on a project in which we compare self-reports of drug use and objective measures of metabolites in hair to validate self-reports. Another problem due to subjective measure of substance use is that we lack information of the potency of the used substance. The fact that we did not see an association between cannabis use and APS could be due to low potency cannabis that was used by our population. However, this seems very unlikely since the presence of high THC/low CBD cannabis is increasing and associations between high potency cannabis and mental health and substance use issues in adolescents have been previously reported (69).

## Conclusion

The past year amount of MDMA use additional to cannabis use was associated with APS among adolescents. Our findings underline the importance to consider MDMA use additional to cannabis when examining the relationships of cannabis use and psychosis risk. Clinicians should be aware of the effects of MDMA use and trauma history to identify individuals among help seeking adolescents with SUDs in order to implement medical treatment of PDs in treatment plans of SUD.

## Data Availability Statement

Due to the nature of this research, participants of this study did not agree for their data to be shared publicly, so supporting data is not available. Please direct any enquiries to the corresponding author.

## Ethics Statement

The studies involving human participants were reviewed and approved by Institutional Review Board of the University Hospital C. G. Carus Dresden. Written informed consent to participate in this study was provided by the participants' legal guardian.

## Author Contributions

MW analyzed the data and wrote the manuscript. SK-P participated in writing the manuscript, data analysis, and contributed to the discussion. LB and VR participated in writing the manuscript and contributed to discussion. YG designed the study, participated in writing the manuscript and contributed to discussion. All authors contributed to the article and approved the submitted version.

## Funding

This study was funded by the Sächsische Aufbaubank-Förderbank (Grant 100362999 to YG).

## Conflict of Interest

SK-P reports personal fees during the past 36 months from Mabuse Verlag, and a one-time lecture honoraria from a consortium of conference sponsors (Janssen-Cilag, Lilly Germany, Novartis Pharma, Pfizer Pharma). VR has received payment for consulting and writing activities from Lilly, Novartis, and Shire Pharmaceuticals, lecture honoraria from Lilly, Novartis, Shire Pharmaceuticals/Takeda, and Medice Pharma, and support for research from Shire Pharmaceuticals/Takeda and Novartis. He has carried out or is currently carrying out clinical trials in cooperation with the Novartis, Shire Pharmaceuticals/Takeda, Servier and Otsuka companies. The remaining authors declare that the research was conducted in the absence of any commercial or financial relationships that could be construed as a potential conflict of interest.

## Publisher's Note

All claims expressed in this article are solely those of the authors and do not necessarily represent those of their affiliated organizations, or those of the publisher, the editors and the reviewers. Any product that may be evaluated in this article, or claim that may be made by its manufacturer, is not guaranteed or endorsed by the publisher.

## References

[1] Trotman HD Holtzman CW Ryan AT Shapiro DI MacDonald AN Goulding SM. The Development of Psychotic Disorders in Adolescence: a potential role for hormones. Horm Behav. (2013) 64:411–9. 10.1016/j.yhbeh.2013.02.018 23998682 PMC4070947

[2] EMCDDA, (n,.d.). Statistical Bulletin 2019—Prevalence of Drug Use. Available online at: http://www.emcdda.europa.eu/data/stats2019/gps_en (accessed May 11, 2020).

[3] Waltereit R Uhlmann A Ehrlich S Roessner V. What happened to the concept of adolescence crisis? Eur Child Adolesc Psychiatry. (2020) 29:1617–9. 10.1007/s00787-020-01660-y 33037929 PMC7641928

[4] Barkus E Murray RM. Substance use in adolescence and psychosis: clarifying the relationship. Annu Rev Clin Psychol. (2010) 6:365–89. 10.1146/annurev.clinpsy.121208.131220 20192802

[5] Correll CU Schooler NR. Negative symptoms in schizophrenia: a review and clinical guide for recognition, assessment, and treatment. Neuropsychiatr Dis Treat. (2020) 16:519–34. 10.2147/NDT.S225643 32110026 PMC7041437

[6] Addington J. The prodromal stage of psychotic illness: Observation, detection or intervention? J Psychiatry Neurosci. (2003) 28:93–7. 12670126 PMC161730

[7] Yung AR McGorry PD. The initial prodrome in psychosis: descriptive and qualitative aspects. Aust New Z J Psychiatry. (1996) 30:587–99. 10.3109/00048679609062654 8902166

[8] American Psychiatric Publishing Inc. Diagnostic and Statistical Manual of Mental Disorders: DSM-5TM, 5th ed (pp. xliv, 947) (2013). American Psychiatric Publishing, Inc.

[9] Addington J Farris M Stowkowy J Santesteban-Echarri O Metzak P Kalathil MS. Predictors of transition to psychosis in individuals at clinical high risk. Curr Psychiatry Rep. (2019) 21:39. 10.1007/s11920-019-1027-y 31037392

[10] Fusar-Poli P Salazar de Pablo G Correll CU Meyer-Lindenberg A Millan MJ Borgwardt S. Prevention of psychosis: advances in detection, prognosis, and intervention. JAMA Psychiatry. (2020) 77:755–65. 10.1001/jamapsychiatry.2019.4779 32159746

[11] Yung AR Yung AR Pan Yuen H Mcgorry PD Phillips LJ Kelly D. Mapping the onset of psychosis: the comprehensive assessment of at-risk mental states. Aust New Zealand J Psychiatry. (2005) 39:964–71. 10.1080/j.1440-1614.2005.01714.x 16343296

[12] Brunette MF Mueser KT Babbin S Meyer-Kalos P Rosenheck R Correll CU. Demographic and clinical correlates of substance use disorders in first episode psychosis. Schizophr Res. (2018) 194:4–12. 10.1016/j.schres.2017.06.039 28697856

[13] Carney R Yung AR Amminger GP Bradshaw T Glozier N Hermens DF. Substance use in youth at risk for psychosis. Schizophr Res. (2017) 181:23–9. 10.1016/j.schres.2016.08.026 27590573

[14] Smith MJ Thirthalli J Abdallah AB Murray RM Cottler LB. Prevalence of psychotic symptoms in substance users: a comparison across substances. Compr Psychiatry. (2009) 50:245–50. 10.1016/j.comppsych.2008.07.009 19374969 PMC2743957

[15] Marconi A Di Forti M Lewis CM Murray RM Vassos E. Meta-analysis of the association between the level of cannabis use and risk of psychosis. Schizophr Bull. (2016) 42:1262–9. 10.1093/schbul/sbw003 26884547 PMC4988731

[16] Kraan T Velthorst E Koenders L Zwaart K Ising HK van den Berg D. Cannabis use and transition to psychosis in individuals at ultra-high risk: review and meta-analysis. Psychol Med. (2016) 46:673–81. 10.1017/S0033291715002329 26568030

[17] Davis GP Compton MT Wang S Levin FR Blanco C. Association between cannabis use, psychosis, and schizotypal personality disorder: findings from the National Epidemiologic Survey of alcohol and related conditions. Schizophrenia Res. (2013) 151:197–202. 10.1016/j.schres.2013.10.018 24200416 PMC3877688

[18] Passie T Benzenhöfer U. MDA, MDMA, and other “mescaline-like” substances in the US military's search for a truth drug (1940s to 1960s). Drug Test Anal. (2018) 10:72–80. 10.1002/dta.2292 28851034

[19] Olszewski D Matias J Monshouwer K Kokkevi A. Polydrug use among 15- to 16-year olds: similarities and differences in Europe. Drugs Educ Prev Policy. (2010) 17:287–302. 10.3109/09687630902806715

[20] Patel A Moreland T Haq F Siddiqui F Mikul M Qadir H. Persistent psychosis after a single ingestion of “Ecstasy” (MDMA). Prim Care Comp CNS Disord. (2011) 13:11l01200. 10.4088/PCC.11l01200 22454798 PMC3304680

[21] Van Kampen J Katz M. Persistent psychosis after a single ingestion of ‘Ecstasy’. *Psychosomatics*. (2001) 42:525–7. 10.1176/appi.psy.42.6.525 11815690

[22] Virani S Daya GN Brainch N Kotapati VP Zaveri D Ahmed S. Persistent psychosis due to single dose of ecstasy. Cureus. (2018) 10:e3058. 10.7759/cureus.3058 30280055 PMC6166901

[23] Lee J Thwaites S Gogos A van den Buuse M. Pharmacological mechanisms involved in sensory gating disruption induced by (±)-3,4-methylene- dioxymethamphetamine (MDMA): relevance to schizophrenia. Brain Sci. (2020) 10:44. 10.3390/brainsci10010044 31941052 PMC7016806

[24] Thwaites SJ Gogos A Van den Buuse M. Schizophrenia-like disruptions of sensory gating by serotonin receptor stimulation in rats: effect of MDMA, DOI and 8-OH-DPAT. Pharmacol Biochem Behav. (2013) 112:71–7. 10.1016/j.pbb.2013.09.016 24120765

[25] Kim HK. Neurophysiological biomarkers in schizophrenia—P50, mismatch negativity, and TMS-EMG and TMS-EEG. Front Psychiatry. (2020) 11:11. 10.3389/fpsyt.2020.00795 32848953 PMC7426515

[26] McKetin R Hickey K Devlin K Lawrence K. The risk of psychotic symptoms associated with recreational methamphetamine use. Drug Alcohol Rev. (2010) 29:358–63. 10.1111/j.1465-3362.2009.00160.x 20636650

[27] McKetin R Lubman DI Baker AL Dawe S Ali RL. Dose-related psychotic symptoms in chronic methamphetamine users: evidence from a prospective longitudinal study. JAMA Psychiatry. (2013) 70:319–24. 10.1001/jamapsychiatry.2013.283 23303471

[28] Bramness JG Rognli EB. Psychosis induced by amphetamines. Curr Opin Psychiatry. (2016) 29:236–41. 10.1097/YCO.0000000000000254 27175554

[29] Hermens DF Lubman DI Ward PB Naismith SL Hickie IB. Amphetamine psychosis: a model for studying the onset and course of psychosis. Med J Aust. (2009) 190:S22–5. 10.5694/j.1326-5377.2009.tb02370.x 19220169

[30] Vaiva G Bailly D Boss V Thomas P Lestavel P Goudemand M. A case of acute psychotic episode after a single dose of ecstasy. L'Encephale. (2001) 27:198–202. 11407274

[31] Stilo SA Murray RM. Non-genetic factors in schizophrenia. Curr Psychiatry Rep. (2019) 21:1. 10.1007/s11920-019-1091-3 31522306 PMC6745031

[32] Cawson P Wattam C Brooker S Kelly G. Child Maltreatment in the United Kingdom: A Study of the Prevalence of Abuse and Neglect. London: NSPCC (2000), p. 21.

[33] Morgan C Fisher H. Environment and schizophrenia: environmental factors in schizophrenia: childhood trauma—a critical review. Schizophr Bull. (2007) 33:3–10. 10.1093/schbul/sbl053 17105965 PMC2632300

[34] Read J Os van J Morrison AP Ross CA. Childhood trauma, psychosis and schizophrenia: a literature review with theoretical and clinical implications. Acta Psychiatr Scand. (2005) 112:330–50. 10.1111/j.1600-0447.2005.00634.x 16223421

[35] Cannon M Jones PB Murray RM. Obstetric complications and schizophrenia: historical and meta-analytic review. Am J Psychiatry. (2002) 159:1080–92. 10.1176/appi.ajp.159.7.1080 12091183

[36] Dalman C Thomas HV David AS Gentz J Lewis G Allebeck P. Signs of asphyxia at birth and risk of schizophrenia: population-based case–control study. Br J Psychiatry. (2001) 179:403–8. 10.1192/bjp.179.5.403 11689395

[37] Hunt GE Large MM Cleary M Lai HMX Saunders JB. Prevalence of comorbid substance use in schizophrenia spectrum disorders in community and clinical settings, 1990–2017: systematic review and meta-analysis. Drug Alcohol Depend. (2018) 191:234–58. 10.1016/j.drugalcdep.2018.07.011 30153606

[38] Addington J Epstein I Liu L French P Boydell KM Zipursky RB. randomized controlled trial of cognitive behavioral therapy for individuals at clinical high risk of psychosis. Schizophr Res. (2011) 125:54–61. 10.1016/j.schres.2010.10.015 21074974

[39] Schultze-Lutter F Klosterkötter J Ruhrmann S. Improving the clinical prediction of psychosis by combining ultra-high risk criteria and cognitive basic symptoms. Schizophr Res. (2014) 154:100–6. 10.1016/j.schres.2014.02.010 24613572

[40] Loewy RL Bearden CE Johnson JK Raine A Cannon TD. The prodromal questionnaire (PQ): preliminary validation of a self-report screening measure for prodromal and psychotic syndromes. Schizophr Res. (2005) 79:117–25. 10.1016/j.schres.2005.03.007 16276559

[41] Ising HK Veling W Loewy RL Rietveld MW Rietdijk J Dragt S. The validity of the 16-item version of the prodromal questionnaire (PQ-16) to screen for ultra high risk of developing psychosis in the general help-seeking population. Schizophr Bull. (2012) 38:1288–96. 10.1093/schbul/sbs068 22516147 PMC3713086

[42] Steinberg AM Brymer MJ Decker KB Pynoos RS. The University of California at Los Angeles post-traumatic stress disorder reaction index. Curr Psychiatry Rep. (2004) 6:96–100. 10.1007/s11920-004-0048-2 15038911

[43] Ruf M Schauer M Elbert T. UPID: UCLA PTSD Index for DSM IV (Child version, revision 1, deutsche Fassung). (pp. 468–472) (2011). Available online at: https://kops.uni-konstanz.de/handle/123456789/18103.

[44] Steinberg AM Brymer MJ Kim S Briggs EC Ippen CG Ostrowski SA. Psychometric properties of the UCLA PTSD reaction Index: part I. J Trauma Stress. (2013) 26:1–9. 10.1002/jts.21780 23417873

[45] Sheehan DV Janavs J. Mini international Neuropsychiatric Interview for Children/Adolescents (MINI Kid). Tampa: University of South Florida, College of Medicine (1998).

[46] Sheehan DV Sheehan KH Shytle RD Janavs J Bannon Y Rogers JE. Reliability and validity of the Mini International Neuropsychiatric Interview for Children and Adolescents (MINI-KID). J Clin Psychiatry. (2010) 71:313–26. 10.4088/JCP.09m05305whi 20331933

[47] Cohen J. Statistical Power Analysis for the Behavioral Sciences (2. ed., reprint). Psychology Press (2009).

[48] Kim JH. Multicollinearity and misleading statistical results. Korean J Anesthesiol. (2019) 72:558–69. 10.4097/kja.19087 31304696 PMC6900425

[49] Duman B Sedes N Baskak B. Additive effects of former methylenedioxymethamphetamine and cannabis use on subclinical psychotic symptoms. Arch Neuropsychiatry. (2017) 54:38–42. 10.5152/npa.2017.16964 28566957 PMC5439470

[50] Thompson E Kline E Ellman LM Mittal V Reeves GM Schiffman J. Emotional and behavioral symptomatology reported by help-seeking youth at clinical high-risk for psychosis. Schizophr Res. (2015) 162:79–85. 10.1016/j.schres.2015.01.023 25638728

[51] Gerstenberg M Hauser M Al-Jadiri A Sheridan EM Kishimoto T Borenstein Y. Frequency and correlates of DSM-5 attenuated psychosis syndrome in a sample of adolescent inpatients with nonpsychotic psychiatric disorders. J Clin Psychiatry. (2015) 76:9435. 10.4088/JCP.14m09435 26646040

[52] Moore THM Zammit S Lingford-Hughes A Barnes TRE Jones PB Burke M. Cannabis use and risk of psychotic or affective mental health outcomes: a systematic review. Lancet. (2007) 370:10. 10.1016/S0140-6736(07)61162-3 17662880

[53] Jones JD Calkins ME Scott JC Bach EC. Gur RE. Cannabis use, polysubstance use, and psychosis spectrum symptoms in a community-based sample of US youth. J Adolescent Health. (2017) 60:653–9. 10.1016/j.jadohealth.2017.01.006 28318911 PMC5441952

[54] Bechtold J Hipwell A Lewis D Loeber R Pardini D. Concurrent and sustained cumulative effects of adolescent marijuana use on subclinical psychotic symptoms. Am J Psychiatry. (2016) 173:781–9. 10.1176/appi.ajp.2016.15070878 27138587 PMC5390686

[55] Dam NTV Earleywine M DiGiacomo G. Polydrug use, cannabis, psychosis-like symptoms. Human Psychopharmacol Clin Exp. (2008) 23:475–85. 10.1002/hup.950 18449850

[56] Alemany S Arias B Fatjó-Vilas M Villa H Moya J Ibáñez MI. Psychosis-inducing effects of cannabis are related to both childhood abuse and COMT genotypes. Acta Psychiatr Scand. (2014) 129:54–62. 10.1111/acps.12108 23445265

[57] Cougnard A Marcelis M Myin-Germeys I De Graaf R Vollebergh W Krabbendam L Lieb R Wittchen H-U Henquet C Spauwen J Van Os J. Does normal developmental expression of psychosis combine with environmental risk to cause persistence of psychosis? A psychosis proneness–persistence model. Psychol Med. (2007) 37:513. 10.1017/S0033291706009731 17288646

[58] Gage SH Hickman M Zammit S. Association between cannabis and psychosis: epidemiologic evidence. Biol Psychiatry. (2016) 79:549–56. 10.1016/j.biopsych.2015.08.001 26386480

[59] Agorastos A Pervanidou P Chrousos GP Kolaitis G. Early life stress and trauma: developmental neuroendocrine aspects of prolonged stress system dysregulation. Hormones. (2018) 17:507–20. 10.1007/s42000-018-0065-x 30280316

[60] Dahoun T Nour MM McCutcheon RA Adams RA Bloomfield MAP Howes OD. The relationship between childhood trauma, dopamine release and dexamphetamine-induced positive psychotic symptoms: a [11C]-(+)-PHNO PET study. Transl Psychiatry. (2019) 9:627. 10.1038/s41398-019-0627-y 31712556 PMC6848217

[61] de la Torre R Farré M Roset PN Pizarro N Abanades S Segura M. Human Pharmacology of MDMA: pharmacokinetics, metabolism, and disposition. Therap Drug Monitor. (2004) 26:137–144. 10.1097/00007691-200404000-00009 15228154

[62] Vegting Y Reneman L Booij J. The effects of ecstasy on neurotransmitter systems: a review on the findings of molecular imaging studies. Psychopharmacology. (2016) 233:3473–501. 10.1007/s00213-016-4396-5 27568200 PMC5021729

[63] Gyongyosi N Balogh B Katai Z Molnar E Laufer R Tekes K. Activation of 5-HT3 receptors leads to altered responses 6 months after MDMA treatment. J Neural Transm. (2010) 117:285–92. 10.1007/s00702-009-0357-z 20052506

[64] Lanteri C Doucet EL Hernández Vallejo SJ Godeheu G Bobadilla A-C. Repeated exposure to MDMA triggers long-term plasticity of noradrenergic and serotonergic neurons. Molecular Psychiatry. (2014) 19:823–33. 10.1038/mp.2013.97 23958955

[65] Ibarra-Lecue I Mollinedo-Gajate I Meana JJ Callado LF Diez-Alarcia R Urigüen L. Chronic cannabis promotes pro-hallucinogenic signaling of 5-HT2A receptors through Akt/mTOR pathway. Neuropsychopharmacology. (2018) 43:2028–35. 10.1038/s41386-018-0076-y 29748632 PMC6098160

[66] Stahl SM. Beyond the dopamine hypothesis of schizophrenia to three neural networks of psychosis: dopamine, serotonin, and glutamate. CNS Spectr. (2018) 23:187–91. 10.1017/S1092852918001013 29954475

[67] Kapur S. Psychosis as a state of aberrant salience: a framework linking biology, phenomenology, and pharmacology in schizophrenia. Am J Psychiatry. (2003) 160:13–23. 10.1176/appi.ajp.160.1.13 12505794

[68] Kelly TM Daley DC. Integrated treatment of substance use and psychiatric disorders. Soc Work Public Health. (2013) 28:388–406. 10.1080/19371918.2013.774673 23731427 PMC3753025

[69] Hines LA Freeman TP Gage SH Zammit S Hickman M Cannon M. Association of high-potency cannabis use with mental health and substance use in adolescence. JAMA Psychiatry. (2020) 77:1044–51. 10.1001/jamapsychiatry.2020.1035 32459328 PMC7254445

