# Supplementary material for: Attenuated Psychotic Symptoms in Adolescents With Chronic Cannabis and MDMA Use
Source: Front Psychiatry. 2022 Jan 21;12:696133. doi: 10.3389/fpsyt.2021.696133 (PMC8814345; doi:10.3389/fpsyt.2021.696133)
Supplement: Supplementary file 1 [file Table_1.docx]

Supplementary Material

## Supplementary Figures

Table S1. Summary of Hierarchical Multiple Regression Analysis for variables of trauma history, birth complications, gender, cannabis and additional MDMA use as well as co-occurring psychiatric disorders as measures predicting APS (*n* = 31).

|  | | Model 1 | | | Model 2 | | | Model 3 | | | Model 4 | | | |
| --- | --- | --- | --- | --- | --- | --- | --- | --- | --- | --- | --- | --- | --- | --- |
| Variable | | Standardized Regression Coefficient *B^1^* | *p*-value^1^ | 95% CI^1^ | Standardized Regression Coefficient *B^1^* | *p*-value^1^ | 95% CI^1^ | Standardized Regression Coefficient *B^1^* | *p*-value^1^ | 95% CI^1^ | Standardized Regression Coefficient *B*^1^ | *p*-value^1^ | 95% CI1 |  |
| *Control variables* | |  | | |  | | |  | | |  |  |  | |
|  | Birth complications | -0.60 | .119 | [-1.32, 0.20] | -1.04 | .094 | [-2.75, 0.04] | -0.81 | .212 | [-2.66, 0.37] | -0.74 | .412 | [-2.76, 1.11] |  |
|  | Gender | 0.38 | .786 | [-2.72, 3.26] | 0.47 | .756 | [-2.65, 3.37] | -0.38 | .740 | [-2.68, 1.71] | -3.28 | .155 | [-7.89, 0.95] |  |
|  | Trauma history | 1.29 | .007 | [0.57, 2.21] | 1.28 | .006 | [0.54, 2.18] | 0.83 | .040 | [0.24, 1.68] | 0.40 | .403 | [-0.49, 1.62] |  |
| *Average amount of past year substance use* | |  | | |  | | |  | | |  |  |  | |
|  | Cannabis use (in grams)  cannabis time since fist use  cannabis time since fist use |  |  |  | 0.31 | .213 | [-0.09, 1.55] | 0.09 | .735 | [-0.68, 0.97] | -0.08 | .818 | [-1.36, 1.02] |  |
|  | MDMA use additional to cannabis (in pills) |  |  |  |  |  |  | 4.73 | .017 | [0.94, 7.73] | 5.34 | .023 | [1.50, 9.40] |  |
| *Co-occurring psychiatric disorders* | |  | | |  | | |  | | |  | | | |
|  | Alcohol Use Disorder |  |  |  |  |  |  |  |  |  | -0.30 | .802 | [-2.11, 2.41] |  |
|  | Affective disorder |  |  |  |  |  |  |  |  |  | 4.47 | .161 | [-2.53, 9.10] |  |
|  | Anxiety disorders |  |  |  |  |  |  |  |  |  | -1.34 | .469 | [-6.56, 2.80] |  |
|  | Post Traumatic Stress Disorder |  |  |  |  |  |  |  |  |  | 1.25 | .608 | [-3.72, 7.14] |  |
|  | Conduct Disorder |  |  |  |  |  |  |  |  |  | 1.14 | .444 | [-2.06, 4.76] |  |
| *Test statistic* | |  | | |  | | |  | | |  | | | |
|  | Corrected *R^2^* | .41 | | | .40 | | | .61 | | | .67 | | | |
|  | *F* (*p*-value) | 7.82 (<.001) | | | 5.98 (<.001) | | | 10.37 (<.001) | | | 7.15 (<.001) | | | |
|  | Δ*R^2^* | .47 | | | .01 | | | .20 | | | .11 | | | |
|  | Δ*F* | 7.82 (<.001) | | | 0.70 (.410) | | | 15.04 (<.001) | | | 1.95 (.131) | | | |

*Note.* MDMA = 3,4‐Methylenedioxymethamphetamine. CI = Confidence Interval. ^1^ = boostrapped values. Substance use variables display the amount of self-rated cannabis or MDMA use per calendar day during the last 12 months.
